# Energy Harvesting Thermoelectric Generators Manufactured Using the Complementary Metal Oxide Semiconductor Process

**DOI:** 10.3390/s130202359

**Published:** 2013-02-08

**Authors:** Ming-Zhi Yang, Chyan-Chyi Wu, Ching-Liang Dai, Wen-Jung Tsai

**Affiliations:** 1 Department of Mechanical Engineering, National Chung Hsing University, Taichung 402, Taiwan; E-Mails: d099061005@mail.nchu.edu.tw (M.-Z.Y.); g9861043@mail.nchu.edu.tw (W.-J.T.); 2 Department of Mechanical and Electro-Mechanical Engineering, Tamkang University, Tamsui 251, Taiwan; E-Mail: ccwu@mail.tku.edu.tw

**Keywords:** energy harvesting, thermoelectric generator, thermocouple, CMOS

## Abstract

This paper presents the fabrication and characterization of energy harvesting thermoelectric micro generators using the commercial complementary metal oxide semiconductor (CMOS) process. The micro generator consists of 33 thermocouples in series. Thermocouple materials are p-type and n-type polysilicon since they have a large Seebeck coefficient difference. The output power of the micro generator depends on the temperature difference in the hot and cold parts of the thermocouples. In order to increase this temperature difference, the hot part of the thermocouples is suspended to reduce heat-sinking. The micro generator needs a post-CMOS process to release the suspended structures of hot part, which the post-process includes an anisotropic dry etching to etch the sacrificial oxide layer and an isotropic dry etching to remove the silicon substrate. Experiments show that the output power of the micro generator is 9.4 μW at a temperature difference of 15 K.

## 1. Introduction

Thermoelectric micro generators can convert waste heat into electrical power to achieve waste energy recycling, and they can be applied in electronic devices providing additional power. The advantages of micro generators fabricated by microelectromechanical system (MEMS) technology include small volume and high efficiency [1]. Several studies have recently employed this technology to develop thermoelectric generators. For instance, Glatz *et al.* [2] used a microfabrication process to manufacture flexible thermoelectric micro generators. The thermoelectric materials in the generators were p- and n-type Bi_2_Te_3_ deposited on a polymer mold by an electrochemical process. The thermoelectric generators had a power factor of 0.29 μW cm^−2^ K^−2^. Lee and Xie [3] presented a thermoelectric power generator using solder-based wafer bonding technology. Three pieces of wafers were bonded to form a vacuum packaged thermoelectric power generator. The output power per area of the generator was derived as 68.6 μW/cm^2^ at the temperature difference of 6 K between two ends of thermocouple junctions. The thermoelectric generators, proposed by Huesgen *et al.* [4], were manufactured using a combined surface and bulk micromachining process. The thermocouples were deposited by thin-film processes with high integration density on the wafer surface. Power factors of 8.14 × 10^−3^ μW mm^−2^ K^−2^ were achieved with thermopiles made of p-Bi_0.5_Sb_1.5_Te_3_ and n-Bi_0.87_Sb_0.13_. Wang *et al.* [5] employed bulk micromachining to produce a thermoelectric generator with the thermocouples made of poly-Si or poly-SiGe, and the generator had a power factor of 1.73 × 10^−2^ μW cm^−2^ K^−2^. Su *et al.* [6] developed a micromachined thermoelectric energy harvester with 6 μm high polycrystalline silicon germanium thermocouples fabricated on a 6 inch wafer. The thermoelectric energy harvester had an output power of 0.4 μW at the temperature difference of 3.5 K. Nishibori *et al.* [7] utilized bulk-Si wet etching to make a micromachined thermoelectric power generator with a ceramic catalyst combustor, and a thermopile of 20 thin-film couples of B-doped Si_0.8_Ge_0.2_/Au and a Pt-loaded alumina ceramic thick-film catalyst combustor were integrated on a thin dielectric membrane. The generator could generate a power of 0.26 μW with a fuel gas flow of 3 vol. % H_2_ in air, 1,000 ccm, at room temperature. Strasser *et al.* [8] adopted surface micromachining to fabricate a thermoelectric micro generator for converting waste heat into electrical power, and the thermoelectric materials in the generator were poly-Si_0.7_Ge_0.3_ and poly-Si.

Fabrication of MEMS devices using the commercial CMOS process is called CMOS-MEMS technique [9,10], and microdevices manufactured by this technique usually need a post-process to release the suspended structures [11,12] or to add the functional films [13,14]. Because the COMS-MEMS technique is compatible with the commercial CMOS process, micro generators have a potential to combine with integrated circuits on-a-chip if they are produced by this technique. Therefore, in this work a thermoelectric micro generator is developed using the CMOS-MEMS technique. The micro generator is composed of 33 thermocouples in series. The hot part of the thermocouples is designed as suspended structures in order to reduce heat-sinking. The post-process of the generator utilizes an anisotropic dry etching to remove the sacrificial oxide layer and an isotropic dry etching to etch the silicon substrate.

## 2. Structure of the Thermoelectric Generator

**Figure 1.**
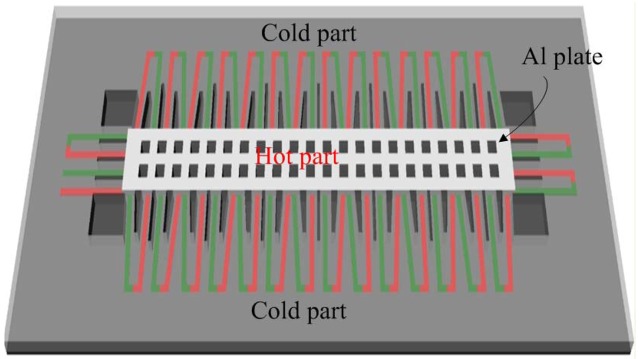
Schematic structure of the energy harvesting thermoelectric generator.

Figure 1 illustrates schematic structure of the energy harvesting thermoelectric micro generator. Area of the generator is about 1,000 × 300 μm^2^. The thermoelectric generator is constructed by 33 thermocouples in series. Each thermocouple is composed of n-type polysilicon and p-type polysilicon strips. The junctions of n-type and p-type polysilicon strips located on the suspended plate are the hot part of the thermocouples, and the other junctions of n-type and p-type polysilicon strips anchored on the silicon substrate are the cold part of the thermocouples. The output power of the generator depends on the temperature difference between the hot and cold parts. The hot part is suspended for reducing heat sink. Dimensions of each polysilicon strip are 120 μm long, 8 μm wide and 0.2 μm thick.

The output voltage of the energy harvesting thermoelectric generator can be expressed as [8]:

(1) $$V_{\text{out}}=n(\alpha_{1}-\alpha_{2})(T_{h}‐T_{c})$$

where *V_out_* represents the output voltage of the thermoelectric generator; *n* is the number of thermocouples in series; *α*_1_ is the Seebeck coefficient of p-type polysilicon; *α*_2_ is the Seebeck coefficient of n-type polysilicon; *T_h_* is the temperature of the hot junctions in thermocouples and *T_c_* is the temperature of the cold junctions in thermocouple. The output voltage of the micro generator depends on the Seebeck coefficients of *α*_1_ and *α*_2_, and it is proportional to the number of thermocouples and the temperature difference of *T_h_* and *T_c_*.

**Figure 2.**
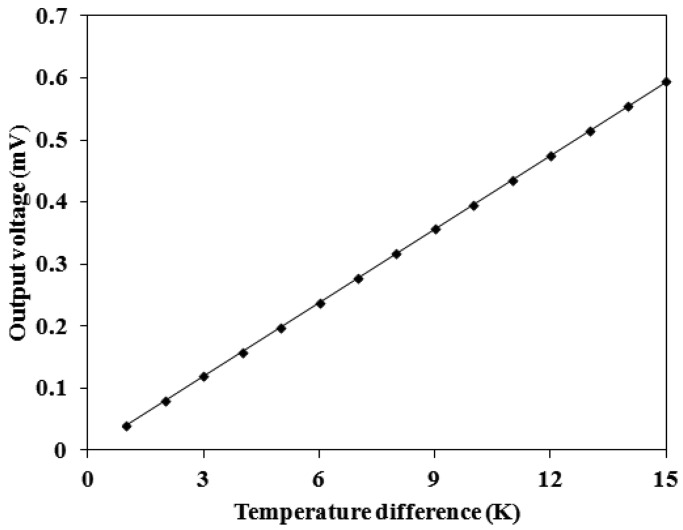
Simulated results of the output voltage for the thermoelectric generator.

In this thermoelectric generator, the parameters are *n* = 33 and *α*_1_*− α*_2_ = 0.0012 mV/K. The value of *α*_1_*− α*_2_ is obtained by testing a test-key thermocouple. Substituting the parameters into Equation (1), the relation between the output voltage and temperature difference for the generator can be obtained. Figure 2 shows the simulated results of the output voltage for the energy harvesting thermoelectric generator. The results show that the thermoelectric generator has an output voltage of about 0.2 mV at the temperature difference of 5 K and an output voltage of about 0.6 mV at the temperature difference of 15 K.

**Figure 3.**
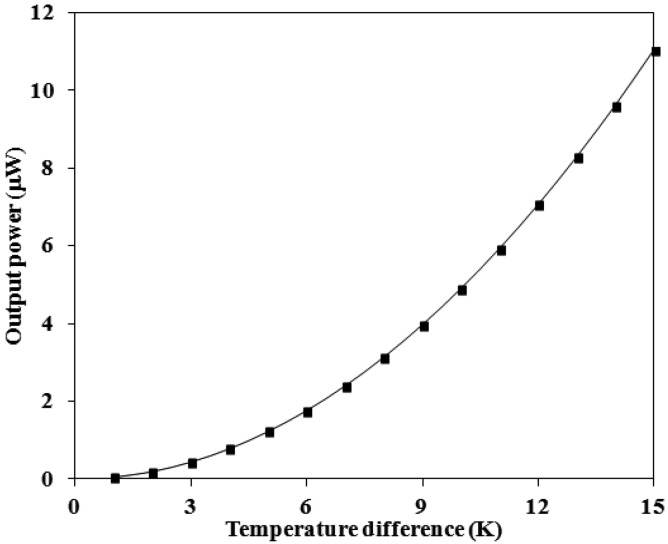
Simulated results of the output power for the thermoelectric generator.

If the external load of the thermoelectric generator equals to the internal load of the thermoelectric generator, the maximum output power of the thermoelectric generator is given by [15]:

(2) $$P_{\max}=\frac{V_{\text{out}}^{2}}{4R}$$

where *P_out_* represents the output power of the energy harvesting thermoelectric generator; *V_out_* is the output voltage of the thermoelectric generator and *R* is the resistance of the thermoelectric generator. In this design, the parameters are *n* = 33, *α*_1_*-α*_2_ = 0.0012 mV/K and *R* = 8 kΩ. Substituting the parameters into Equations (1) and (2), the maximum output power of the energy harvesting thermoelectric generator can be obtained. Figure 3 shows the simulated results of maximum output power for the thermoelectric generator. The results depicts that the output power of the energy harvesting thermoelectric generator is about 1.22 μW at the temperature difference of 5 K and about 11.03 μW at the temperature difference of 15 K.

## 3. Fabrication of the Thermoelectric Generator

**Figure 4.**
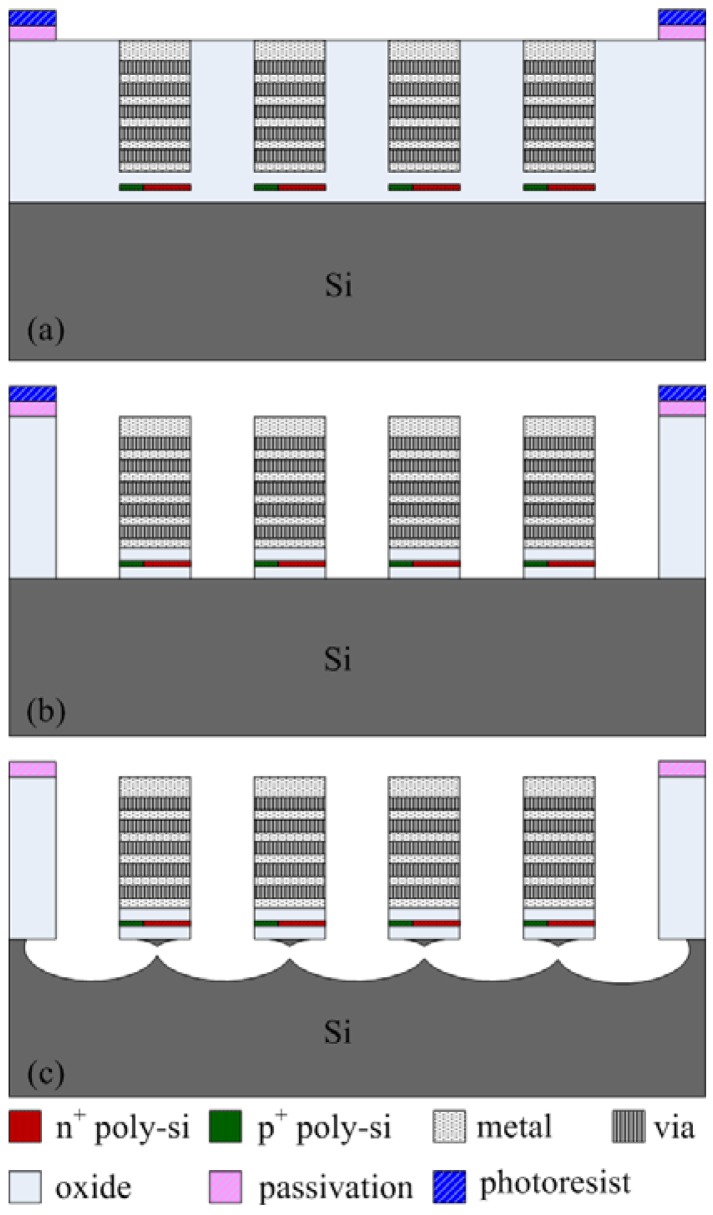
Fabrication flow of the thermoelectric generator, (**a**) after completion of the CMOS process; (**b**) etching the oxide sacrificial layer; (**c**) etching the silicon substrate.

The commercial 0.35 μm CMOS process of Taiwan Semiconductor Manufacturing Company (TSMC) was utilized to fabricate the energy harvesting thermoelectric generator. Figure 4 presents the fabrication flow of the energy harvesting thermoelectric generator. Figure 4(a) illustrates the cross-section of the thermoelectric generator after completion of the CMOS process. The thermoelectric generator required a post-process to release the suspended structures of the hot part in order to reduce heat-sinking of the hot part.

**Figure 5.**
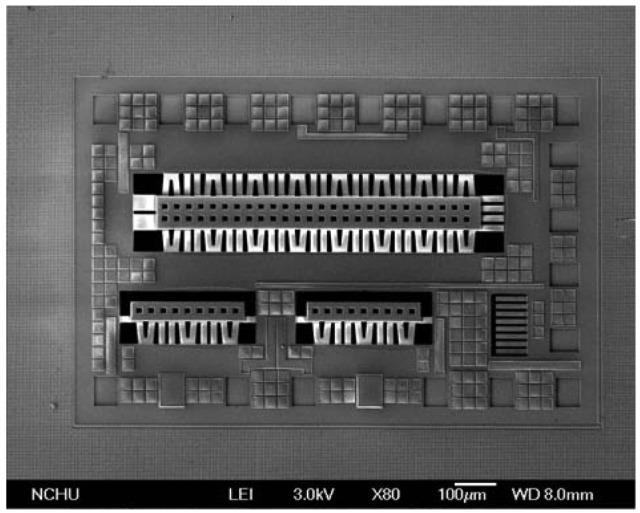
SEM image of the thermoelectric generator.

**Figure 6.**
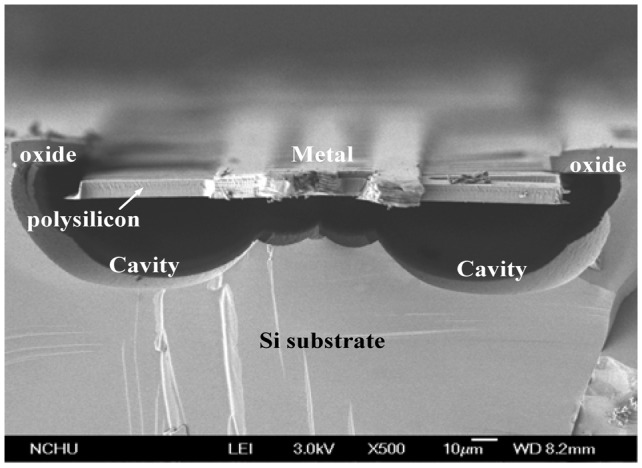
SEM image of cross-sectional view for the thermoelectric generator.

The post-processing involves two steps: one removes silicon dioxide and the other etches the silicon substrate. Figure 4(b) shows that the silicon dioxide is removed. Reactive ion etching (RIE) with CHF_3_/O_2_ is used to etch the sacrificial oxide layer, and to expose the silicon substrate. Figure 4(c) shows that the silicon substrate is removed. An RIE dry etching with XeF_2_ is employed to etch the silicon substrate, and to obtain the suspended structure of hot part in the generator. Figure 5 displays a scanning electron microscope (SEM) image of the energy harvesting thermoelectric generator after the post-process. In order to understand the suspended plate released condition, the thermoelectric generator was cut along its cross-section. Figure 6 shows an SEM image of cross-sectional view for the energy harvesting thermoelectric generator. The suspended plat in the generator was released completely.

## 4. Results and Discussion

**Figure 7.**
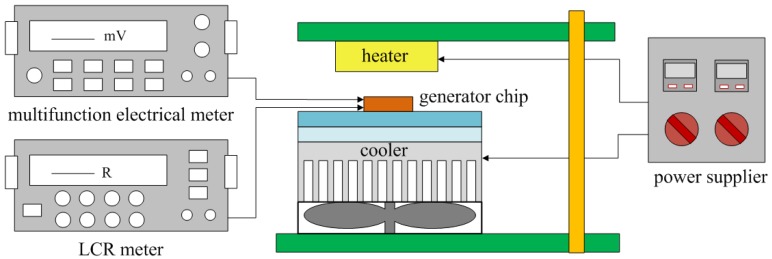
Measurement setup of the thermoelectric generator.

The performance of the energy harvesting thermoelectric generator was measured by a heater, a cooler, an LCR meter and a multifunction electrical meter. Figure 7 shows the measurement setup of the thermoelectric generator. The heater provided a heat source to the thermoelectric generator, and the cooler was used to increase a heat sink for the cold part in the generator.

**Figure 8.**
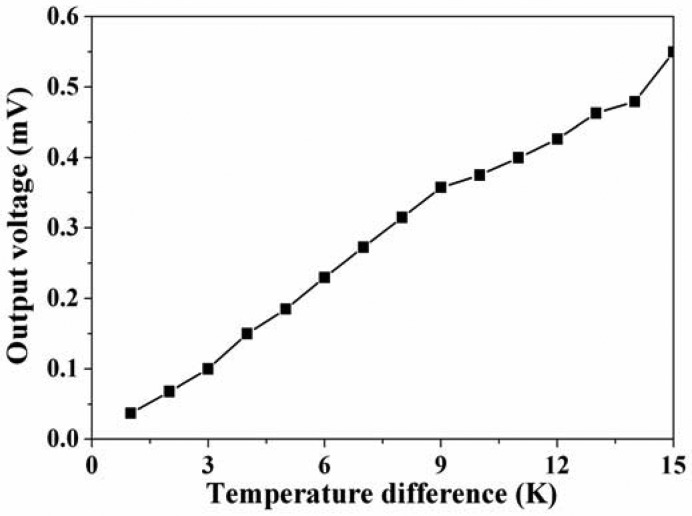
Measured results of the output voltage for the thermoelectric generator.

An infrared thermometer detected the temperature difference between the hot and cold parts in the thermoelectric generator. The LCR meter was used to measure the resistance of the thermoelectric generator, and the measured results showed that the resistance of the generator was 8 kΩ. The multifunction electrical meter recorded the output voltage of the thermoelectric generator. Figure 8 shows the measured results of the output voltage for the thermoelectric generator. The results depicted that the output voltage of the thermoelectric generator was about 0.18 mV at the temperature difference of 5 K and about 0.55 mV at the temperature difference of 15 K. The output voltage per area of the thermoelectric generator was about 12.1 mV mm^−2^ K^−1^.

**Figure 9.**
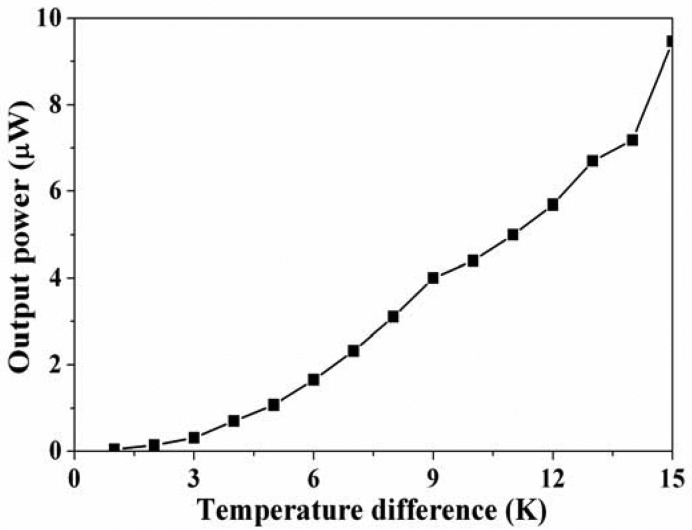
Measured results of the output power for the thermoelectric generator.

The output power of the thermoelectric generator was evaluated by its output voltage. Substituting the measured output voltage in Figure 8 and the resistance *R* = 8 kΩ into Equation (2), the maximum output power of the thermoelectric generator was obtained. Figure 9 shows the maximum output power of the energy harvesting thermoelectric generator. The results showed that the output power of the thermoelectric generator was about 1.1 μW as the temperature difference of 5 K and was about 9.4 μW as the temperature difference of 15 K. The power factor of the thermoelectric generator was 1.4 × 10^−2^ μW mm^−2^ K^−2^. A comparison with the simulated results in Figure 3, the simulated output power was 11.03 μW at the temperature difference of 15 K, so the percentage of error was about 14%, in which resulted from the evaluated error of Seebeck coefficient in the thermocouples.

Kao *et al.* [16] developed a thermoelectric micro generator fabricated using the CMOS-MEMS technique, and it had an output voltage per area of 0.093 mV mm^−2^ K^−1^ and a power factor of 6.4 × 10^−7^ μW mm^−2^ K^−2^. Huesgen *et al.* [3] proposed a thermoelectric micro generator with a power factor of 8.14 × 10^−3^ μW mm^−2^ K^−2^. A comparison to Huesgen *et al.* [4] and Kao *et al.* [16], indicates that the output power factor in this work exceeds that of Kao *et al.* [16]. Because area of the generator is reduced and its thermocouple number is increased, the generator of this work has higher output power factor than that of Kao *et al.* [16].

## 5. Conclusions

An energy harvesting thermoelectric micro generator manufactured using the commercial CMOS-process has been implemented. The micro generator was constructed by 33 thermocouples in series. Each thermocouple consisted of p-type and n-type polysilicon strips. The output power of the generator depended on the temperature difference between the hot and cold parts in the thermocouples. The hot part of the generator located on a suspended plate for reducing the heat-sinking. A post-CMOS process was utilized to release the hot part of the generator, and the post-process included an anisotropic dry etching with CHF_3_/O_2_ RIE to remove the sacrificial oxide layer and an isotropic dry etching with XeF_2_ RIE to etch the silicon substrate. The experimental results showed that the output power of the thermoelectric generator was about 9.4 μW as the temperature difference of 15 K and the power factor of the generator was 1.4 × 10^−2^ μW mm^−2^ K^−2^. The thermoelectric micro generator is suitable for application in microelectronic devices as an auxiliary electrical power source. The power of the device can be enhanced using many generators in series. In addition, the power of the generator can also be improved by structure design to increase the temperature difference of hot and cold junctions and by changing thermocouple materials to increase the Seebeck coefficient difference.
